# Clinical performance validation of an integrated cartridge based bedside blood gas analyzer system in the acute care setting

**DOI:** 10.1038/s41598-025-06710-6

**Published:** 2025-07-01

**Authors:** Lixia Yang, Dazhi Chi, Cuixiang Zhen, Wenjie Zhang, Junhua Yang, Jinlian Shao, Hua Xie

**Affiliations:** 1 Obstetrics and Gynecology Medical Center, Zhujiang Hospital, Southern Medical University, No. 253 Middle Industrial Road, Haizhu District, Guangzhou, 510280 China; 2 Emergency Department, Zhujiang Hospital, Southern Medical University, No. 253 Middle Industrial Road, Haizhu District, Guangzhou, 510280 China

**Keywords:** Diagnostic markers, Endocrine system and metabolic diseases, Outcomes research

## Abstract

This study aims to evaluate the clinical performance of the EG-i30 blood gas analyzer and its test cartridge (EG10+), a maintenance-free electrochemical point-of-care blood gas analyzer system (referred to as EG), in comparison with the established ABL90 FLEX blood gas analyzer system (referred to as ABL) in the acute care setting. A total of 216 clinical residual samples from 94 patients were analyzed using both systems. Key blood gas and electrolyte parameters, including pH, *p*O_2_, *p*CO_2_, K^+^, Na^+^, iCa^2+^, Cl^−^, Lac, Glu, and Hct, were measured. Performance was assessed using statistical methods including Bland-Altman plots, Pearson’s correlation, concordance correlation coefficient (CCC), and Passing-Bablok regression. ROC curve analysis evaluated the EG’s diagnostic performance for hyperlactatemia, hypokalemia, and hyperkalemia. The EG demonstrated excellent correlation with the ABL, with r values ranging from 0.969 to 0.992 and CCC values between 0.958 and 0.991 for all parameters. Bias analysis at the medical decision levels showed that all parameters were within the allowable error limits. ROC analysis for Lac and K^+^ revealed high sensitivity and specificity. The EG provides reliable and accurate blood gas analysis comparable to the ABL, offering a portable, cost-effective solution for rapid diagnostics in emergency and critical care settings.

**Supplementary Information:**

The online version contains supplementary material available at 10.1038/s41598-025-06710-6.

## Introduction

Blood gas analysis (BGA) is a critical diagnostic tool used to measure key parameters in human blood, including the partial pressure of oxygen (*p*O_2_), partial pressure of carbon dioxide (*p*CO_2_), and pH. These parameters provide valuable insights into respiratory function, oxygenation status, and acid-base balance, enabling healthcare professionals to understand the physiological state of patients^1–3^. BGA is essential for diagnosing and differentiating conditions such as hypoxemia, respiratory failure, and acid-base disturbances^3–5^. It also plays a vital role in the management of surgical procedures, emergency care, and intensive monitoring in critical care settings^6–8^.

The increasing demand for point-of-care testing (POCT) devices in emergency and critical care settings has driven the development of a wide range of blood gas analyzers. These devices vary in their underlying technologies, reagents, and quality control materials, leading to differences in performance^9–12^. Such variability in test results can directly impact clinical decision-making, underlining the need to ensure the accuracy, reliability, and consistency of these systems to safeguard both patient safety and diagnostic precision^13–15^.

Recent advances in electrochemical sensor technology have addressed some of these challenges, improving both accuracy and portability while reducing maintenance requirements. Cartridge-based systems, such as the Eaglenos blood gas analyzer system (referred to as EG), which includes the EG-i30 analyzer and the EG10 + test cartridge, simplify the design and operational complexity, potentially improving reliability and portability in acute care settings.

Despite these technological improvements, rigorous validation data comparing newer cartridge-based systems to established laboratory analyzers remain limited. The Clinical and Laboratory Standards Institute (CLSI) EP09-A3 guideline underscores the necessity of standardized method comparisons to ensure device reliability in real-world clinical practice^16^.

This study adheres to the CLSI EP09-A3 guideline to evaluate the analytical performance of the EG system across ten critical parameters: pH, *p*CO_2_, *p*O_2_, potassium (K^+^), sodium (Na^+^), ionized calcium (iCa^2+^), chloride (Cl^−^), lactate (Lac), glucose (Glu), and hematocrit (Hct). Using residual clinical blood gas samples from routine diagnostic testing, the study benchmarks the EG system’s performance against the established ABL90 blood gas analyzer system (referred to as ABL) to assess its suitability for use in acute care settings (Table 1).

## Result

### Outliers detection and analysis

The distribution of test results for all parameters is presented in Table 2. In this study, a total of ten parameters were analyzed, and outlier detection was performed for each. Outliers were identified for four parameters: *p*O_2_ (1 outlier), K^+^ (4 outliers), Na^+^ (3 outliers), and Lac (3 outliers). The remaining six parameters showed no indications of outliers. All identified outliers were documented and included in the dataset.

**Table 1:** Results of consistency and correlation analyses between EG-i30 and ABL90.

Parameters	Result Distribution Range				Bland-Altman Analysis			CCC	Passing-Bablok Regression		Linear model validity	*r*
	Maximum	Minimum	Mean	Median	Mean +1.96SD	Mean	Mean -1.96SD		Slope (95% CI)	Intercept (95% CI)		
pH	7.668	6.919	7.413	7.407	0.02763	-0.001093	-0.02982	0.9868	1.0175 (0.9895 to 1.0517)	-0.1300 (-0.3833 to 0.07787)	*P*=0.73	0.9868
*p*CO_2_ (mmHg)	97.6	10.1	36.3	37.2	3.1306	-0.1032	-3.3370	0.9894	1.0152 (0.9980 to 1.0420)	-0.5538 (-1.5696 to 0.2000)	*P*=0.51	0.9895
*p*O_2_ (mmHg)	245	30	111.5	114.8	10.4294%	1.9747%	-6.4800%	0.9848	1.0000 (0.9823 to 1.0275)	2.0000 (-0.4482 to 3.7458)	*P*=0.58	0.9866
K^+^ (mmol/L)	8.2	2.2	3.8	3.9	0.2174	0.001852	0.001852	0.9872	1.0000 (1.0000 to 1.0000)	0.0000 (0.0000 to 0.0000)	*P*=0.90	0.9872
Na^+^ (mmol/L)	168	116	142	141.1	-2.4614	0.9722	4.4059	0.9725	1.0345 (1.0000 to 1.0785)	-3.8621 (-10.2185 to 1.0000)	*P*=0.73	0.9798
iCa^2+^ (mmol/L)	1.47	0.33	1.09	1.07	0.05379	0.001111	-0.05157	0.9853	0.9821 (0.9375 to 1.0000)	0.02018 (0.0000 to 0.06768)	*P*=0.33	0.9856
Cl^-^ (mmol/L)	133	83	109	108.7	1.9797	-1.5833	-5.1464	0.9578	0.9375 (0.9000 to 1.0000)	5.3125 (-2.0000 to 9.2000)	*P*=0.14	0.9776
Lac (mmol/L)	20.0	0.5	1.6	2.3	0.8463	0.03750	-0.7713	0.9845	1.0000 (0.9608 to 1.0184)	0.10000 (-2.2204E-16 to 0.1123)	*P*=0.24	0.9846
Glu (mmol/L)	30.0	4.0	9.1	10.0	11.4778%	1.8501%	-7.7777%	0.9909	0.9703 0.9474 to 1.0000	0.4743 0.2000 to 0.6714	*P*=0.73	0.9916
Hct (%)	67	11	26	28	16.5960%	0.3908%	-15.8144%	0.9691	1.0000 1.0000 to 1.0435	0.0000 -0.7446 to 0.0000	*P*=0.86	0.9693

**Table 2:** Bias at medical decision level for ten parameters. The allowable total error (TEa) for iCa^2+^ and Lac was derived from the technical standards of comparable products, while tea for the remaining parameters was based on the CLIA PT final rule.

Parameters	MDL	Bias(95% CI)	TEa	Decision	Reference
pH	7.3	-0.002553(-0.006540 to 0.001000 )	± 0.04	Equivalent	CLIA PT final rule
	7.35	-0.001681(-0.004648 to 0.0010000)	± 0.04	Equivalent	CLIA PT final rule
	7.45	0.00006473(-0.003000 to 0.002939 )	± 0.04	Equivalent	CLIA PT final rule
*p*CO_2_ (mmHg)	35.25	-0.01970(-0.2936 to 0.2301)	± 5	Equivalent	CLIA PT final rule
	45	0.1280(-0.2000 to 0.4664 )	± 5	Equivalent	CLIA PT final rule
	50	0.2038(-0.1961 to 0.5986)	± 5	Equivalent	CLIA PT final rule
	69.79	0.72%(-0.29 to 1.98%)	± 8%	Equivalent	CLIA PT final rule
*p*O_2_ (mmHg)	30	2.0000(0.3868 to 3.2711)	± 15	Equivalent	CLIA PT final rule
	60	2.0000(1.1944 to 3.0000)	± 15	Equivalent	CLIA PT final rule
	80	2.0000(1.7217 to 3.0000)	± 15	Equivalent	CLIA PT final rule
	105	1.90%(1.73 to 2.86%)	± 15%	Equivalent	CLIA PT final rule
K^+^ (mmol/L)	3.0	0.0000(0.0000 to 0.0000)	± 0.3	Equivalent	CLIA PT final rule
	5.8	0.0000(0.0000 to 0.0000)	± 0.3	Equivalent	CLIA PT final rule
	7.5	0.0000(0.0000 to 0.0000)	± 0.3	Equivalent	CLIA PT final rule
Na^+^ (mmol/L)	115	0.1034(-1.1633 to 1.0000)	± 4	Equivalent	CLIA PT final rule
	135	0.7931(0.3913 to 1.0000)	± 4	Equivalent	CLIA PT final rule
	150	1.3103(1.0000 to 1.6603)	± 4	Equivalent	CLIA PT final rule
iCa^2+^ (mmol/L)	0.37	0.01357(0.0000 to 0.04467)	± 5	Equivalent	Benchmark
	3.30	-1.17%(-4.25 to 0.30%)	± 5%	Equivalent	Benchmark
Cl^-^ (mmol/L)	90	-0.35%(-2.22 to 0.42%)	± 5%	Equivalent	CLIA PT final rule
	112	-0.35%(-2.22 to 0.42%)	± 5%	Equivalent	CLIA PT final rule
Lac (mmol/L)	0.5	0.10000(-1.1102E-16 to 0.1034)	± 0.6	Equivalent	Benchmark
	1.7	0.10000(-0.0007692 to 0.1000)	± 0.6	Equivalent	Benchmark
	2.0	0.10000(-0.007262 to 0.1000)	± 0.6	Equivalent	Benchmark
	5.0	2.00%(-2.07 to 2.97%)	± 12%	Equivalent	Benchmark
Glu (mmol/L)	6.7	4.11%(2.99 to 5.22%)	± 8%	Equivalent	CLIA PT final rule
	10.0	1.77%(0.89 to 2.88%)	± 8%	Equivalent	CLIA PT final rule
Hct (%)	14	0.00%(-0.97 to 0.00%)	± 4%	Equivalent	CLIA PT final rule
	33	0.00%(0.00 to 1.97%)	± 4%	Equivalent	CLIA PT final rule
	56	0.00%(0.00 to 2.82%)	± 4%	Equivalent	CLIA PT final rule
	70	0.00%(0.00 to 3.16%)	± 4%	Equivalent	CLIA PT final rule

### Consistency and correlation analysis

The results of the consistency and correlation assessment for all parameters are summarized in Table 2. Bland-Altman plots (BA plots), Pearson’s correlation coefficient (r), and concordance correlation coefficient (CCC) were used to evaluate the consistency and correlation between the EG and ABL for each of the ten parameters.

**Fig. 1:**
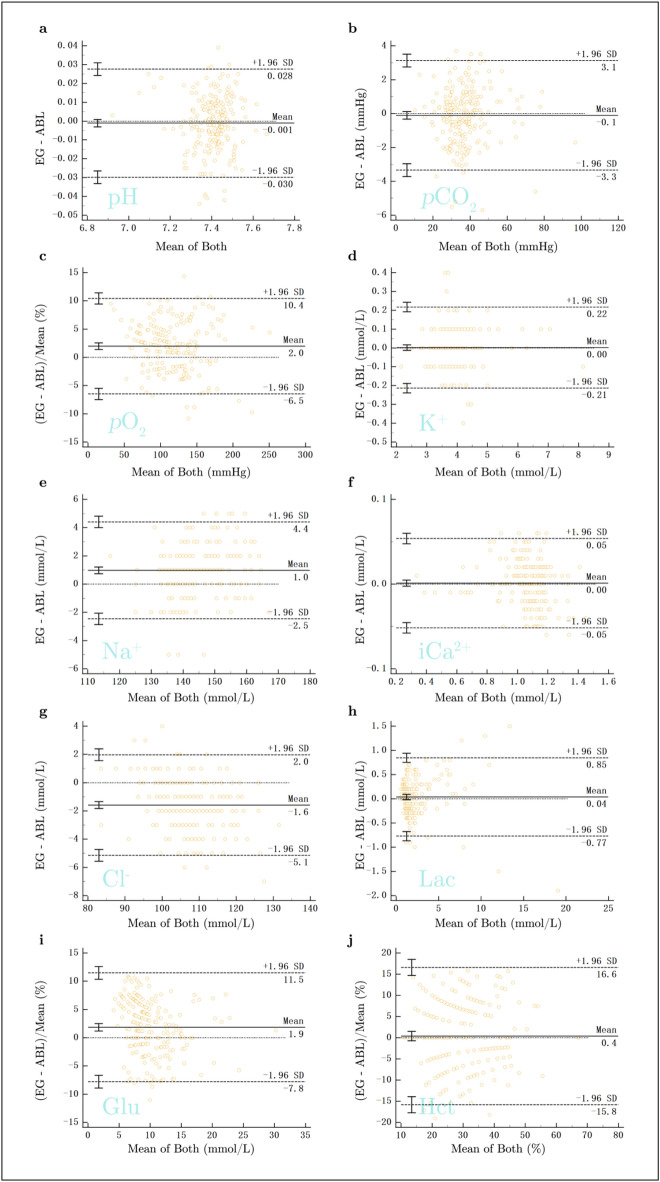
Bland-Altman Plots Demonstrating Agreement Between the EG and ABL. Bland-Altman plots illustrating the agreement between the EG and ABL for the ten clinical parameters: (**a**) pH, (**b**) *p*CO_2_, (**c**) *p*O_2_, (**d**) K^+^, (**e**) Na^+^, (**f**) iCa^2+^, (**g**) Cl^−^, (**h**) Lac, (**i**) Glu, and (**j**) Hct. The plots display the difference between the two systems as a percentage of the average measurement across a range of values. The middle dashed line represents the mean difference, while the upper and lower dashed lines represent the limits of agreement (± 1.96 standard deviations). The mean difference and limits of agreement for each parameter are indicated on the respective plots.

For all parameters, the x-axis of the BA plots represents the mean of the two test results, as shown in Fig. 1. For *p*O_2_, Glu, and Hct, the y-axis is expressed as the relative difference, while for the remaining seven parameters, it represents the absolute difference. The limits of agreement for all ten parameters are presented in the BA plots (Fig. 1). The r values for all ten parameters ranged from 0.969 to 0.992. Similarly, the CCC values ranged from 0.958 to 0.991.

### Regression analysis

**Fig. 2:**
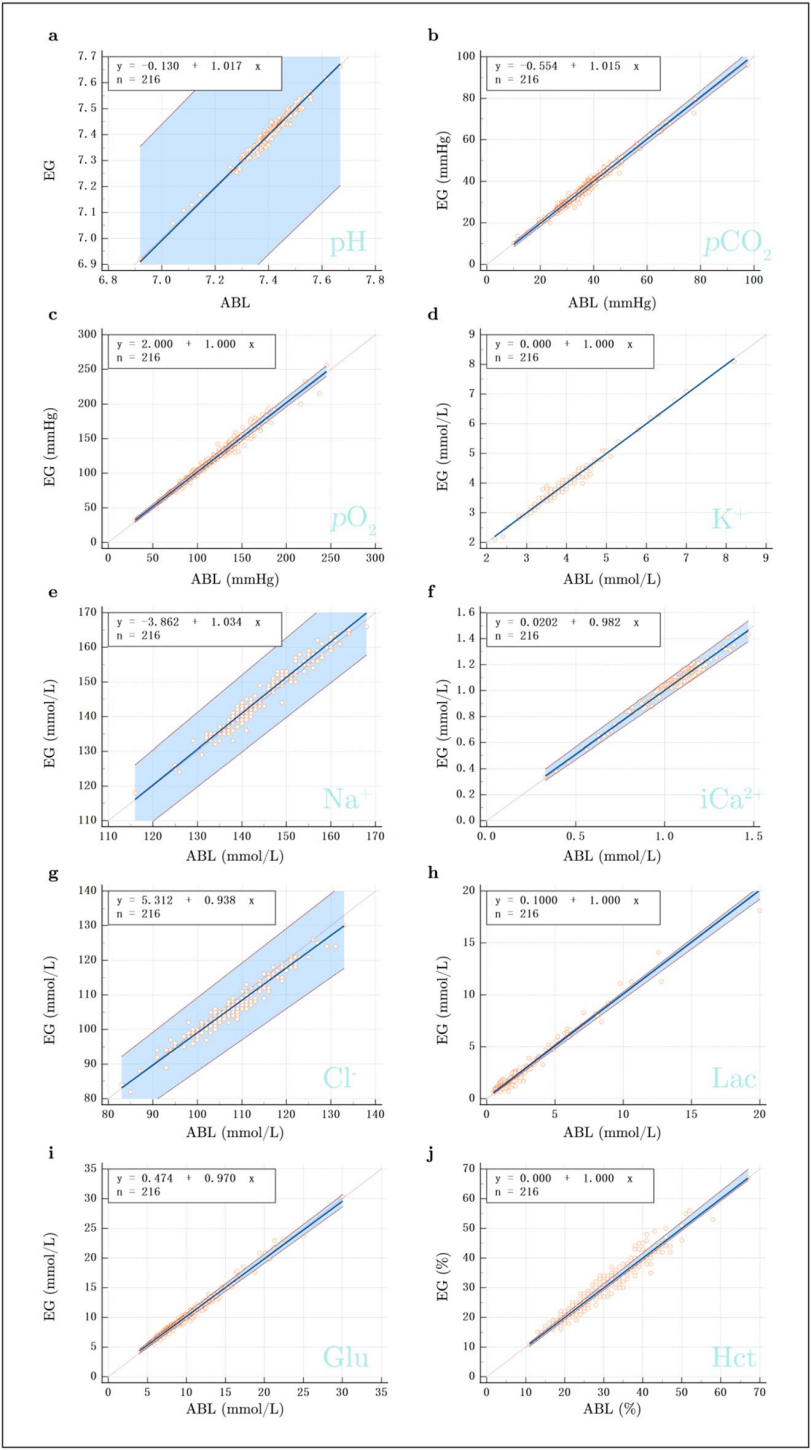
Passing-Bablok Regression Analysis for the Agreement Between the EG and ABL90. Passing - Bablok regression analysis for each of the ten clinical parameters: (**a**) pH, (**b**) *p*CO_2_, (**c**) *p*O_2_, (**d**) K^+^, (**e**) Na^+^, (**f**) iCa^2+^, (**g**) Cl^−^, (**h**) Lac, (**i**) Glu, and (**j**) Hct. The regression lines represent the relationship between the EG and ABL90, with the 95% confidence intervals shown as shaded areas. The equation for each regression line, along with the sample size (*n* = 216), is displayed in the upper-left corner of each plot.

Further analysis of the consistency and linear relationship between the EG and ABL was conducted using Passing-Bablok regression (Fig. 2). The regression slopes and intercepts for each parameter, along with their respective 95% confidence intervals, are presented in Table 2. As shown in the Passing-Bablok regression results, the slopes for all parameters ranged from 0.938 to 1.035, and the intercepts ranged from − 3.862 to 5.313. The validity of the linear model, with p-values greater than 0.05 for all parameters, indicate no significant deviation from the expected linear relationship.

### Bias at medical decision levels (MDLs)

The bias at the MDLs for each of the ten clinical parameters is summarized in Table 3. All parameters fall within the allowable error range, with both the bias and its 95% confidence interval (CI) at the MDLs remaining within the acceptable deviation limits.

**Table 3:** ROC curve analysis of EG-i30 for diagnosing hyperlactatemia, hypokalemia, and hyperkalemia.

Conditions	AUC	Sensitivity	Specificity	Youden index J	Associated criterion	Significance level *P* (Area=0.5)
Hyperlactatemia	0.973	94.37%	89.66%	0.8402	> 1.8	<0.0001
Hypokalemia	0.982	97.62%	91.38%	0.8900	≤ 3.5	<0.0001
Hyperkalemia	0.999	100.00	99.04	0.9904	> 4.8	<0.0001

### Receiver operating characteristic (ROC) curve analysis

**Fig. 3:**
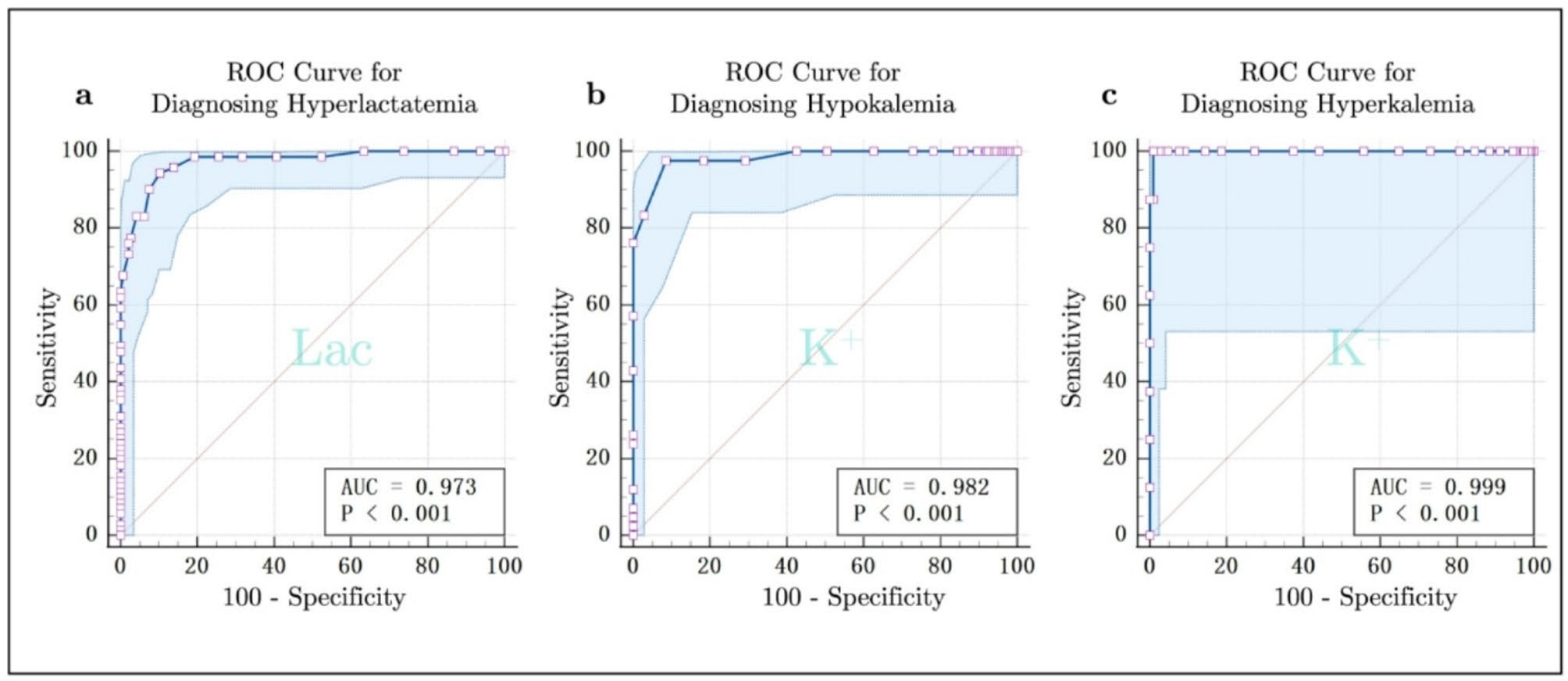
ROC Curve Analyses for Diagnosing Hyperlactatemia, Hypokalemia, and Hyperkalemia. ROC curves for Lac in diagnosing hyperlactatemia (**a**), and for K^+^ in diagnosing hypokalemia (**b**) and hyperkalemia (**c**). The AUC and P-value results for each parameter are displayed in the respective panels.

The diagnostic performance of the EG was assessed using ROC curve analysis for two key parameters: Lac for diagnosing hyperlactatemia, and K⁺for diagnosing both hypokalemia and hyperkalemia. The results are summarized in Table 4, with the corresponding ROC curves presented in Fig. 3.

**Table 4:** Comparison of technical characteristics between EG-i30 and ABL90.

Characteristic		EG-i30 (EG10+)	ABL90 FLEX
Size(Width×Depth×Height)		235 mm×210 mm×160 mm	250 mm×290 mm×470 mm
Weight		3±0.5 kg	<12 kg
Calibration Method/frequency		Automatic, completed with the testing process	Automatic/Manual, performed regularly;
Measurement Principles	Potentiometry	pH, *p*CO_2_, K^+^, Na^+^, iCa^2+^, Cl^-^	
	Amperometry	Glu, Lac, *p*O_2_	Glu, Lac
	Optical measurement	/	*p*O_2_
	AC impedance method	Hct	/
	spectrophotometry	/	Hb (estimated Hct)
Indicated Range	pH	6.500 to 8.000	6.300 to 8.000
	*p*O_2_ (mmHg)	10 to 700	0 to 800
	*p*CO_2_ (mmHg)	10 to 150	5 to 250
	K^+^ (mmol/L)	2.0 to 9.0	0.5 to 25.0
	Na^+^ (mmol/L)	100 to 180	7 to 350
	iCa^2+^ (mmol/L)	0.25 to 2.50	0.1 to 9.99
	Cl^-^ (mmol/L)	65 to 140	7 to 350
	Lac (mmol/L)	0.50 to 20.00	-0.1-31.0
	Glu (mmol/L)	1.1 to 38.0	0 to 60
	Hct (%)	10 to 70	0 to 83.3(estimate)

The ROC curve analysis for diagnosing hyperlactatemia, hypokalemia, and hyperkalemia showed the following results: For hyperlactatemia (*n* = 71), the Youden index was 0.840, and the AUC was 0.973 (95% CI: 0.942 to 0.990). For hypokalemia (*n* = 42), the Youden index was 0.890, with an AUC of 0.982 (95% CI: 0.954 to 0.995). For hyperkalemia (*n* = 8), the Youden index was 0.990, and the AUC was 0.999 (95% CI: 0.981 to 1.000). All three analyses had P-values < 0.001.

## Discussion

This study provides the first evaluation of the EG system using clinical samples, demonstrating its high analytical performance across ten key parameters (pH, *p*CO_2_, *p*O_2_, K^+^, Na^+^, iCa^2+^, Cl^−^, Lac, Glu, and Hct) compared to the ABL90. Statistical analyses, including Bland-Altman plots, Pearson correlation, and CCC, confirmed strong consistency between the EG and ABL systems, with r values and CCCs exceeding 0.95 for all parameters. Passing-Bablok regression analysis further validated these results, demonstrating robust linear relationships between the two systems, with slopes approaching 1 and intercepts near 0. The test results covered a wide physiological and pathological range, with biases at medical decision levels (MDLs) remaining within acceptable standards, further supporting the EG system’s reliability. ROC curve analysis, using the ABL as the reference standard, demonstrated that the EG system achieved high sensitivity and specificity in detecting elevated lactate levels (> 2 mmol/L) and potassium abnormalities (hypokalemia and hyperkalemia), with AUC values exceeding 0.97 across these conditions.

As the first study assessing the EG system’s clinical performance, this evaluation establishes a reference for POCT blood gas analyzers. Previous assessments of the i-STAT system, a comparable POCT device, showed strong agreement with the Radiometer ABL 800 Flex for pH and lactate (R^2^ = 0.90 and 0.96, respectively), alongside moderate correlations for *p*O_2_, *p*CO_2_, BE, and HCO_3_^−^ (R^2^ = 0.70 to 0.92), in septic patients, supporting its utility in critical care settings; however, its evaluation was limited to fewer parameters^13^. Similarly, a study of the epoc analyzer reported high correlations (R² > 0.94) with the Siemens Rapidlab 1265 for pH, *p*CO_2_, *p*O_2_, hemoglobin, K^+^, Glu, and Lac, but lower correlations for Na^+^ (R² = 0.82) and iCa^2+^(R^2^ = 0.87), and with the Dimension Vista for Na^+^ (R² = 0.73) and K^+^ (R² = 0.89)^12^. In contrast, the EG system exhibited consistently high correlation (r and CCC > 0.95) across all ten parameters against the ABL90, with no notable systematic biases. These findings indicate reliability comparable to established POCT systems, with enhanced consistency for Na^+^ and iCa^2+^.

The EG system’s overall performance across ten key parameters shows potential in clinical settings requiring rapid diagnostics, such as emergency departments and intensive care units. Literature on POCT devices indicates their potential to shorten decision-making time in pre-hospital care, thereby improving efficiency in time-critical scenarios^17,18^. In disaster response, POCT devices enable rapid assessment of hemoglobin and lactate levels to guide transfusions and reduce complications^19^. Compact devices like the EG could potentially support such applications in resource-limited settings. Additionally, integrating POCT into emergency logistics can improve rescue efficiency in disaster-affected areas^20^a benefit that could be supported by the miniaturized design of devices like the EG. Future studies should validate its long-term stability and inter-device reproducibility to confirm its clinical utility.

Certain limitations should be acknowledged in this evaluation. First, outliers identified in the Bland-Altman analysis were retained for the final evaluation. A detailed analysis showed these outliers were near the performance acceptance criteria for the respective parameters, suggesting they were close to, but not necessarily within, the acceptable range. Retaining them reflects real-world variability and avoids overestimating the EG system’s performance; however, this may obscure potential biases at medical decision levels, necessitating further studies with refined outlier handling. Second, the diagnostic performance for potassium abnormalities (hypokalemia and hyperkalemia) was assessed using the ABL90 as the reference standard, without central laboratory biochemical assays. As central laboratory methods are the gold standard, this limits confidence in the EG’s accuracy for detecting critical potassium imbalances. Future studies with central laboratory comparisons could confirm its diagnostic accuracy.

In conclusion, this study confirms the EG system’s high analytical consistency across ten key parameters compared to the ABL90, highlighting its potential as a reliable point-of-care blood gas analyzer for critical care and resource-limited settings. POCT devices like the EG hold promise for applications in scenarios such as pre-hospital emergency care and disaster response, but further studies are warranted to establish their diagnostic accuracy and clinical reliability in these settings.

## Materials and methods

### Sample source and size

This study utilized arterial whole blood samples collected from patients in the Emergency Department and Emergency Intensive Care Unit (EICU) of Zhujiang Hospital, Southern Medical University, between December 2024 and January 2025. Inclusion criteria: Patients requiring arterial blood gas analysis as part of clinical care. Exclusion criteria: (1) History of blood-borne infections (e.g., hepatitis B, syphilis, HIV); (2) Delayed initiation of testing (> 2 min post-collection); (3) Sample contamination or insufficient volume (< 0.5 mL); (4) Other conditions deemed ineligible by investigators.

A total of 216 residual samples from 94 patients were included. Ethical approval for this study was granted by the Medical Ethics Committee of Zhujiang Hospital, Southern Medical University (Ethics Approval No: 2024-KY-408-01). The study was registered with ClinicalTrials.gov under the Identifier: NCT06726473. Informed consent was obtained from all participants and/or their legal guardians prior to inclusion in the study. All methods were performed in accordance with relevant ethical guidelines and regulations, and ethical standards were maintained throughout the study.

According to the guideline, clinical laboratories must test at least 40 samples within the measurement range when introducing a new procedure^[17]^. This study validates ten parameters simultaneously, so we increased the sample size fivefold to ensure broad coverage of the testing range.

### Main instruments and reagents

The experimental measurements were conducted using the EG-i30 blood gas analyzer (Eaglenos Sciences, Inc., Nanjing, China; International model: EN102), which was recently introduced in the Emergency Medicine Department at Zhujiang Hospital, Southern Medical University, along with the EG10 + test cartridge (Lot Number: 24111201). Reference measurements were performed using the ABL90 FLEX blood gas analyzer (Radiometer Medical ApS, Brønshøj, Denmark) and its corresponding reagent kits, which are routinely accredited and utilized in our department. Relevant detailed characteristic and comparisons are shown in Table 4.

### Sample collection and handling

Arterial blood was collected using 3 mL arterial blood gas syringes with an inner wall coating of 50U lithium-zinc balanced heparin (Arterial Blood Gas Sampler, Model 3302; Westmed Inc., USA). The arterial puncture sites included the radial, brachial, and femoral arteries. Arterial blood specimens collected from the same patient at different time points were treated as independent samples. Routine analysis with the ABL was performed within 2 min of collection at room temperature (22–25 °C). Residual samples were immediately transferred to the EG and analyzed within 1 min. No storage or transportation steps were involved. Due to the limited volume of residual samples and potential post-sample metabolic changes, we performed a single measurement of the residual samples, in accordance with the EP09-A3 guideline.

### Quality control

Standardized procedures were employed during sample collection and analysis to minimize pre-analytical variability^2^. Samples with visible hemolysis, clots, or insufficient volume (< 0.5 mL) were excluded. All syringes were pre-filled with balanced lithium-zinc heparin. Both analyzers underwent daily quality control using manufacturer-specified protocols:

- EG: EG10 + cartridges (Lot 24111201) with dedicated QC solution (Lot 24111201).
- ABL: Automated QC mode with S9030/S9040/S9050 (Lot 7593339).

### Study parameters

The EG analyzer directly measures ten parameters: pH, *p*O_2_, *p*CO_2_, K^+^, Na^+^, iCa^2+^, Cl^−^, Lac, Glu, and Hct. These parameters were included in the analysis, with measurements performed using both the EG and ABL analyzers for comparison. Calculated parameters, such as bicarbonate (HCO_3_^−^), base excess (BE), and total CO_2_, were excluded from this study.

### Statistical methods

All statistical analyses were performed using MedCalc 23.0.1 and GraphPad Prism 10.1.2 software. Outliers were detected using the Extreme Studentized Deviate (ESD) method (α = 0.05) following CLSI EP09-A3 guidelines^16^. Analyses were performed in MedCalc 23.0.1, with flagged outliers reviewed against predefined clinical acceptability thresholds.

For all parameters, Bland-Altman plots were constructed, with the horizontal axis representing the mean of the measurements from the two detection methods. The vertical axis was selected based on the type of bias observed: either the absolute difference or the percentage difference between the two methods, depending on whether the bias was constant Standard Deviation (SD) or proportional Coefficient of Variation (CV).

Absolute bias was calculated as the difference between the EG and ABL measurements: Absolute Bias = *EG* - *ABL*. Relative bias was calculated as the percentage difference between the EG and ABL results, normalized to the mean of the two methods: Relative Bias = ($\:\frac{EG\:-ABL}{Mean\:of\:EG\:and\:ABL}$)×100%. These calculations were performed for all paired measurements to assess the magnitude and direction of systematic deviations between the two systems.

The consistency and correlation between the two testing systems were further assessed using Pearson’s correlation coefficient, the CCC, and Passing-Bablok regression. The bias at the MDLs, along with its corresponding CIs, was estimated using the bootstrap technique. MDL values for K+, Na+, Cl-, Glu, and Hct were derived from Clinical Decision Levels for Laboratory Tests (Statland BE, 1987)^21^. For the remaining parameters, MDLs were determined by the authors based on their clinical relevance and normal reference ranges.

Performance standards were predefined according to the *Clinical Laboratory Improvement Amendments (CLIA) Proficiency Testing Final Rule* (87 FR 2022)^22^. For parameters not explicitly addressed in the final rule, such as iCa^2+^ and Lac, we referred to performance requirements from comparable commercially available devices, notably the Abbott i-STAT system.

ROC curve analysis was performed to evaluate the diagnostic performance of the EG for diagnosing hyperlactatemia, hypokalemia, and hyperkalemia. The ABL served as the reference method for measuring lactate and potassium. Diagnostic thresholds were defined as follows: hyperlactatemia, lactate > 2 mmol/L; hyperkalemia, potassium > 5 mmol/L; and hypokalemia, potassium < 3.5 mmol/L. The area under the curve (AUC) and P-values were used to assess sensitivity, specificity, and overall diagnostic accuracy.

To facilitate calculation, we standardized the precision of the measurement results. Specifically, *p*O_2_ and Hct values from the ABL were rounded to the nearest integer, while Lac values from the EG were rounded up to one decimal place.

## Electronic supplementary material

Below is the link to the electronic supplementary material.

Supplementary Material 1

## Data Availability

The datasets generated and analyzed during the current study are available from the corresponding author upon reasonable request.
